# Protein interactions of the transcription factor Hoxa1

**DOI:** 10.1186/1471-213X-12-29

**Published:** 2012-10-22

**Authors:** Barbara Lambert, Julie Vandeputte, Sophie Remacle, Isabelle Bergiers, Nicolas Simonis, Jean-Claude Twizere, Marc Vidal, René Rezsohazy

**Affiliations:** 1 Molecular and Cellular Animal Embryology group, Life Sciences Institute (ISV), Université Catholique de Louvain, Louvain-la-Neuve, 1348, Belgium; 2 Bioinformatique des Génomes et des Réseaux (BiGRe), Université libre de Bruxelles, Bruxelles, Belgium; 3 GIGA-R and Gembloux Agro Bio-Tech, Université de Liège, Liège, 4000, Belgium; 4 Center for Cancer Systems Biology (CCSB) and Department of Cancer Biology, Dana-Farber Cancer Institute, Boston, MA, 02215, USA; 5 Department of Genetics, Harvard Medical School, Boston, MA, 02115, USA

**Keywords:** Hox, Hoxa1, ORFeome, Interactome

## Abstract

**Background:**

Hox proteins are transcription factors involved in crucial processes during animal development. Their mode of action remains scantily documented. While other families of transcription factors, like Smad or Stat, are known cell signaling transducers, such a function has never been squarely addressed for Hox proteins.

**Results:**

To investigate the mode of action of mammalian Hoxa1, we characterized its interactome by a systematic yeast two-hybrid screening against ~12,200 ORF-derived polypeptides. Fifty nine interactors were identified of which 45 could be confirmed by affinity co-purification in animal cell lines. Many Hoxa1 interactors are proteins involved in cell-signaling transduction, cell adhesion and vesicular trafficking. Forty-one interactions were detectable in live cells by Bimolecular Fluorescence Complementation which revealed distinctive intracellular patterns for these interactions consistent with the selective recruitment of Hoxa1 by subgroups of partner proteins at vesicular, cytoplasmic or nuclear compartments.

**Conclusions:**

The characterization of the Hoxa1 interactome presented here suggests unexplored roles for Hox proteins in cell-to-cell communication and cell physiology.

## Background

The conserved family of homeodomain Hox transcription factors is critically involved in patterning the body plan of bilaterian embryos by controlling multiple morphogenetic and organogenetic processes during animal development [1-4]. Modifications in Hox protein expression and activity have likely contributed to the evolutionary diversification of animal forms [5,6]. Misregulation or mutation of several Hox proteins has been associated with pathologies like cancer or neuropathies [7,8].

Hox proteins are transcription factors which regulate expression of target genes and chromatin remodeling [9]. A handful of proteins that interact with Hox proteins have been identified so far, and these are almost exclusively transcription factors, like the well-characterized Three Amino acid Loop Extension (TALE) homeodomain proteins Pbx/Exd and Prep/Meis/Hth [10], TFIIE*β*[11], TATA Binding Protein (TBP) [12], Gli3 [13], Maf [14], Smad [15,16], High Mobility Group protein 1 (HMG1) [17], or transcriptional coregulators like CREB Binding Protein (CBP)/p300 [18-20]. Hox proteins may also form complexes with the translation initiation factor eIF4E to control the translation of target mRNAs [21]. Some Hox-like homeodomain proteins can be secreted into the extracellular compartment and translocate through the cell membrane to gain access to the cytosol and nucleus of neighboring cells, so it has been proposed that Hox proteins could display a paracrine transcriptional activity [22,23].

Numerous transcription factors, involved in critical developmental processes, like Smad, STAT, β-catenin or NFκB, are primarily signal transducers. Though primarily cytoplasmic, upon activation these can translocate to the nucleus, where they convey signaling by affecting gene regulation. As signal transducers these transcription factors can interact with enzymatically active membrane receptors, adaptor proteins, signal transducing kinases, or ubitiquin ligases. Possibly, Hox transcription factors could similarly fulfill pivotal roles at the heart of developmental processes, acting at the crossroads between cell-to-cell communication and cell fate determination. To our knowledge no exhaustive interaction screen has been performed to detect functional connections for a Hox protein.

Here, we conducted a proteome-wide screening for candidate interactors of Hoxa1. *Hoxa1* is one of the earliest *Hox* genes to be expressed during embryonic development. It is involved in hindbrain segmentation and patterning [1,24,25]. *Hoxa1* misregulation has been associated with mammary carcinogenesis [26]. We used a stringent high-throughput yeast two-hybrid (Y2H) approach to systematically test pairwise combinations, using Hoxa1 both as a bait and as a prey against the human ORFeome v3.1 resource, which contains 12,212 ORFs representing 10,214 genes [27]. Of the 59 Hoxa1 interactions identified, 45 could be validated by *in vivo* affinity binding assays in co-transfected animal cells. A striking subset of the validated interactors are not proteins involved in gene regulation. Rather, these interactors are adaptor proteins or modulators of the Bone Morphogenetic Proteins (BMP)/Tumor Growth Factor (TGF) β, Tumor Necrosis Factor (TNF), Receptor-Tyrosine Kinases (RTK) and integrins signal transduction pathways. Other interactors participate in cell adhesion or endosomal trafficking. We detected 41 interactions in live cells by Bimolecular Fluorescence Complementation (BiFC). Depending on the different proteins identified, interactions either take place in the cytoplasm, in the nucleus, in association with vesicles or show a variable pattern from cell to cell, underscoring a dynamic interplay with Hoxa1. Numerous identified Hoxa1 partners reported to interact with each other within known pathways share similar intracellular patterns of Hoxa1 interaction by BiFC. We conclude that Hoxa1 can contact several subunits of multi-molecular functional platforms involved in cell signaling, cell-adhesion, or cell shape regulation.

## Results

### A proteome-wide yeast two-hybrid screening for Hoxa1 interactors

The yeast two-hybrid (Y2H) is a powerful approach for large-scale screenings to identify binary protein-protein interactions [28,29]. DB-Hoxa1 was tested pairwise against 12,212 open reading frame (ORF)-derived proteins from the human ORFeome version 3.1 [27] fused to the Gal4 activation domain (AD). In this configuration, we detected 40 distinct interactions (Table 1). We also screened in the other configuration, Hoxa1 as a prey (AD-Hoxa1) against the full hORFeome in fusion with the Gal4 DB. In the second configuration we detected 28 interactions, of which 8 were also detected in the DB-Hoxa1/AD-ORFs configuration (Table 2). A total of 59 candidate Hoxa1 interactors were identified. We found the Hoxa1 homodimerization interaction and 8 out of the 9 Hoxa1 interactions, previously described in the literature [28,30] (Table 1 and 2).

**Table 1:** Interaction partners for Hoxa1 revealed by yeast two-hybrid screening using DB-Hoxa1

**Protein symbol**	**Protein name**	**Gene ID**	**UniProtKB/Swiss-Prot**	**Protein function**	**BiFC signal**	**Confirmed by co-purification**
ADAMTSL4 (TSRC1)	ADAMTS-like 4	54507	Q6UY14	TNF-induced apopotosis	Nuclear	Y
BAT2L	HLA-B associated transcript 2-like	84726	Q5JSZ5	Unknown	Nuclear	Y
C1orf94	chromosome 1 open reading frame 94	84970	Q6P1W5	Protein binding	Cytoplasmic	Y
CCDC33	coiled-coil domain containing 33	80125	Q8N5R6	Protein binding	/	N
EFCAB4B	EF-hand calcium binding domain 4B	84766	Q9BSW2	Calcium binding	Nuclear, and vesicular	Y
EFEMP2*^§^	EGF-containing fibulin-like extracellular matrix protein 2	30008	O95967	Fibulin-like, unknown	n.d.	Y
FAM154A	family with sequence similarity 154, member A	158297	Q8IYX7	Unknown	Nuclear, vesicular, cytoplasmic	Y
FHL5 (ACT)	four and a half LIM domains 5	9457	Q5TD97	Transcription factor (zinc finger), and kinesin and actin-binding protein	Nuclear	Y
GPRIN2	G protein regulated inducer of neurite outgrowth 2	9721	O60269	G protein interaction	/	N
HOXA1^§^	homeobox A1	3198	P49639	Transcription factor (homeodomain)	Nuclear	Y
HOXD3	homeobox D3	3232	P31249	Transcription factor (homeodomain)	Nuclear	Y
HSFY1	heat shock transcription factor, Y-linked 1	86614	Q96LI6	Transcription factor (heatshock factor)	/	N
KRTAP26-1^§^	keratin associated protein 26-1	388818	Q6PEX3	Keratin associated	Nuclear	Y
KRTAP3-2^§^	keratin associated protein 3-2	83897	Q9BYR7	Keratin associated	Nuclear	Y
KRTAP3-3^§^	keratin associated protein 3-3	85293	Q9BYR6	Keratin associated	/	N
KRTAP4-12*^§^	keratin associated protein 4-12	83755	Q9BQ66	Keratin associated	Cytoplasmic	Y
LGALS13	lectin, galactoside-binding, soluble, 13	29124	Q9UHV8	Lipase activity, signaling (regulator of protein kinases)	Nuclear, vesicular, cytoplasmic	Y
LNX2	ligand of numb-protein X 2	222484	Q8N448	Molecular scaffold, E3 ubiquitin ligase, signaling regulator (Notch), associated to cell adhesion molecules	Nuclear and cytoplasmic	Y
LPXN*	leupaxin	9404	O60711	Signaling (focal adhesion), Transcription factor	Vesicular and cytoplasmic	Y
MGAT5B (GnT-VB)	mannosyl (α-1,6-)-glycoprotein β-1,6-N-acetyl-glucosaminyltransferase, isozyme B	146664	Q3V5L5	Glycosyltransferase, focal adhesion dynamics	Nuclear	Y
N4BP2L2 (PFAAP5)	NEDD4 binding protein 2-like 2	10443	Q92802	Transcription factor or co-regulator	Nuclear	Y
NR4A1 (Nur77)	nuclear receptor subfamily 4, group A, member 1	3164	P22736	Transcription factor (nuclear hormone receptor)	/	N
OGT	O-linked N-acetylglucosamine (GlcNAc) transferase	8473	O15294	Glycosyltransferase, transcription co-regulator	Nuclear and cytoplasmic	Y
PCSK5^§^	proprotein convertase subtilisin/kexin type 5	5125	Q92824	Pro-protein convertase	/	N
PDLIM7 (LMP-1)	PDZ and LIM domain 7	9260	Q9NR12	Signaling regulator (BMP, IGFBP pathways)	Cytoplasmic	Y
PLSCR1*	phospholipid scramblase 1	5359	O15162	Phospholipid scramblase, signaling regulatior (receptor tyrosine kinases, protein kinases), transcription factor	Nuclear	Y
PLSCR4^§^	phospholipid scramblase 4	57088	Q9NRQ2	Phospholipid scramblase, transcription factor	Nuclear	Y
PRDM14	PR domain containing 14	63978	Q9G2V8	Histone methyltransferase	Nuclear, vesicular, cytoplasmic	Y
RBCK1	RanBP-type and C3HC4-type zinc finger containing 1	10616	Q9BYM8	Signaling regulator (TNFR, protein kinases), ubiquitin ligase, transcription factor	Nuclear, vesicular, cytoplasmic	Y
RBPMS (Hermes)	RNA binding protein with multiple splicing	11030	Q93062	Signaling regulator (TGFβ), RNA binding	Nuclear, vesicular, cytoplasmic	Y
RGS17	regulator of G-protein signaling 17	26575	Q9UGC6	Signaling regulator (G proteins)	/	N
RGS20	regulator of G-protein signaling 20	8601	O76081	Signaling regulator (G proteins, protein kinases)	Nuclear and cytoplasmic	Y
SPRY1	sprouty homolog 1	10252	O43609	Signaling regulator (receptor tyrosine kinases)	Nuclear, vesicular, cytoplasmic	Y
SPRY2	sprouty homolog 2	10253	O43597	Signaling regulator (receptor tyrosine kinases, protein kinases)	/	N
TRAF1	TNF receptor-associated factor 1	7185	Q13077	Signaling regulator (TNFR pathway)	Vesicular and cytoplasmic	Y
TRAF2	TNF receptor-associated factor 2	7186	Q12933	Signaling regulator (TNFR pathway)	Vesicular and cytoplasmic	Y
TRIM23 (ARD1)	tripartite motif-containing 23	373	P36406	Vesicular trafficking and signaling regulator (TNF pathway), E3 ubiquitin ligase	n.d.	Y
TRIP6*	thyroid hormone receptor interactor 6	7205	Q15654	Cytoskeleton and signaling regulator (focal adhesion, TNFR), transcription co-regulator	Nuclear	Y
ZBTB16 (PLZF)	zinc finger and BTB domain containing 16	7704	Q05516	Transcription factor (zinc finger), signaling regulator (GPCR, ProRenin Receptor)	Nuclear and vesicular	Y
ZBTB32 (FAZF)	zinc finger and BTB domain containing 32	27033	Q9Y2Y4	Transcription factor (zinc finger)	Cytoplasmic	Y

* Previously reported by Rual et al., 2005.

^§^ Revealed by both AD-ORF and DB-ORF screening.

^*a*^ Y = yes; N = no.

**Table 2:** Interaction partners for Hoxa1 revealed by Y2H screening using AD-Hoxa1

**Protein symbol**	**Protein name**	**Gene ID**	**UniProtKB/Swiss-Prot**	**Protein function**	**BiFC signal**	**Confirmed by co-purification**
ADCK4	aarF domain containing kinase 4	79934	Q96D53	Ser/Thr kinase	/	N
AGPAT1	1-acylglycerol-3-phosphate O-acyltransferase 1	10554	Q99943	Acetyltransferase	/	N
BSCL2 (Seipin)	Berardinelli-Seip congenital lipodystrophy 2	26580	Q96G97	Unknown	/	N
DKKL1 (Soggy)	dickkopf-like 1	27120	Q9UK85	Signaling modulator (Wnt pathway)	/	N
EFEMP2*^§^	EGF-containing fibulin-like extracellular matrix protein 2	30008	O95967	Fibulin-like, unknown	n.d.	Y
FAM108A1	family with sequence similarity 108, member A1	81926	Q96GS6	unknown	Nuclear	Y
GP9	glycoprotein IX (platelet)	2815	P14770	Multifunctional receptor, cytoskeleton and signaling regulator (integrins, focal adhesion, PI3K)	/	N
GRN*	granulin	2896	P28799	Growth factor, transcription factor (in GRN precursor form)	n.d.	Y
HOXA1^§^	homeobox A1	3198	P49639	Transcription factor (homeodomain)	Nuclear	Y
HSD3B7	hydroxy-delta-5-steroid dehydrogenase, 3 β- and steroid delta-isomerase 7	80270	Q9H2F3	Dehydrogenase	/	N
IKZF2 (Helios)	IKAROS family zinc finger 2	22807	Q9UKS7	Transcription fator (zinc finger)	Nuclear	Y
KRT81	keratin 81	3887	Q14533	Intermediate filament	Nuclear and cytoplasmic	Y
KRTAP26-1^§^	keratin associated protein 26-1	388818	Q6PEX3	Keratin associated	Nuclear	Y
KRTAP3-2^§^	keratin associated protein 3-2	83897	Q9BYR7	Keratin associated	Nuclear	Y
KRTAP3-3^§^	keratin associated protein 3-3	85293	Q9BYR6	Keratin associated	/	N
KRTAP4-12*^§^	keratin associated protein 4-12	83755	Q9BQ66	Keratin associated	Cytoplasmic	Y
KRTAP5-9	keratin associated protein 5-9	3846	P26371	Keratin associated	Vesicular and cytoplasmic	Y
LIMS1 (PINCH1)	LIM and senescent cell antigen-like domains 1	3987	P48059	Cytoskeleton and signaling regulator (focal adhesion, integrins, receptor tyrosine kinases)	Nuclear	Y
MDFI* (I-mfa)	MyoD family inhibitor	4188	Q99750	Signaling regulator (channels, Wnt, JNK pathways) - Transcription factor (I-mfa domain),	Nuclear, vesicular, cytoplasmic	Y
PCSK5^§^	proprotein convertase subtilisin/kexin type 5	5125	Q92824	Pro-protein convertase	/	N
PDCD6IP (Alix)	programmed cell death 6 interacting protein	10015	Q8WUM4	Endosome formation and vesicular trafficking, cytoskeleton and signaling regulator (Focal adhesion, TNFR pathway, EGFR, PDGFR)	Vesicular and cytoplasmic	Y
PFKM	phosphofructokinase, muscle	5213	P08237	Glycolysis	/	N
PITX2	paired-like homeodomain 2	5308	Q99697	Transcription factor (homeodomain)	Nuclear	Y
PLSCR4^§^	phospholipid scramblase 4	57088	Q9NRQ2	Phospholipid scramblase, transcription factor	Nuclear	Y
RAB33A	member RAS oncogene family	9363	Q14088	Small GTPase, vesicular trafficking (Ras pathway)	Nuclear	Y
SMOC1	SPARC related modular calcium binding 1	64093	Q9H4F8	Extracellular matrix protein, signaling, migration and differentiation modulator	n.d.	Y
TRAPPC6A*	trafficking protein particle complex 6A	79090	O75865	Vesicular trafficking	Nuclear	Y
ZZZ3	zinc finger, ZZ-type containing 3	26009	Q8IYH5	Transcription factor (zinc finger)	Nuclear	Y

* Previously reported by Rual et al., 2005.

^§^ Revealed by both AD-ORF and DB-ORF screening.

^*a*^ Y = yes; N = no.

### Co-purification from animal cells validate forty-five Hoxa1 interactors

**Figure 1:**
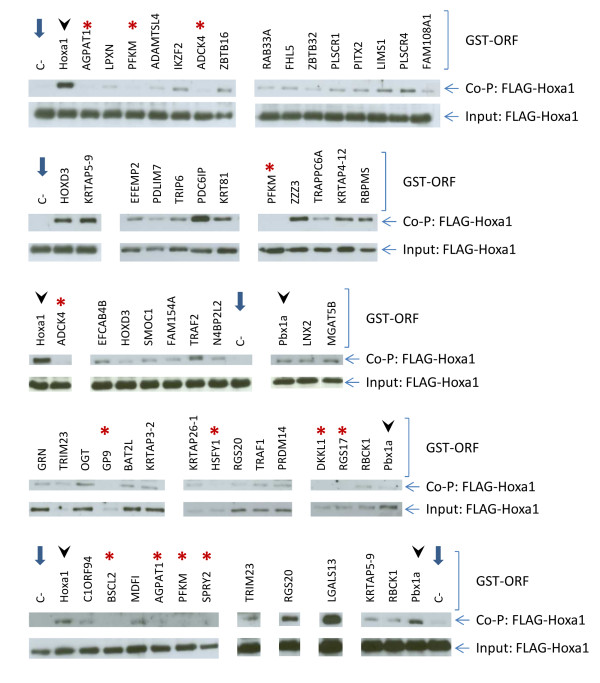
**Validation of 45 out of the 59 interactions revealed for Hoxa1 by affinity co-purification on glutathione-agarose beads.** Candidate interactors were fused with a GST-tag and co-expressed in transfected cells with a FLAG-Hoxa1 fusion protein. Western blots were run to detect FLAG-Hoxa1 from cell extracts before (Input) or after (Co-P) purification. The Hoxa1-Hoxa1 or PBX1A-Hoxa1 interactions were used as positive controls (see lanes with arrowheads). Negative control corresponds to transfected cells with the only FLAG-Hoxa1 fusion protein (C-, lanes with blue arrows). Some interactors which could not be confirmed by co-purification are also shown (red asterisks).

To validate the 59 interactions identified by the Y2H screen by an orthogonal assay we turned to affinity co-purification of a FLAG-Hoxa1 fusion protein co-expressed with glutathione S-transferase (GST)-tagged candidate interactors in transfected COS7 or HEK293T cells. In absence of GST-partners, there was no or very weak background binding of FLAG-Hoxa1 onto the glutathione-agarose beads (Figure 1). As positive controls we measured Hoxa1 dimer formation [30,31] and the reproducible interaction between Hoxa1 and Pbx1a [32] (Figure 1). In total, affinity co-purification from co-transfected cells confirmed 45 out of the 59 Y2H interactors (Table 1 and 2), in the presence of which a detectable amount of FLAG-Hoxa1 remained associated to the GST-fusion/glutathione-agarose beads and could be detected on western blots. It should be noted however that some interactions could not be confirmed because the corresponding GST-ORF fusion was expressed at an undetectable level, if at all (data not shown).

### Bioinformatics functional analysis

To determine if Hoxa1 preferentially targets particular biological functions or pathways, we tested for statistical enrichment in regards to the Gene Ontology GO [33]), Kyoto Encyclopedia of Genes and Genomes KEGG; [34]) and Pathway Commons databases (http://www.pathwaycommons.org).

We observed that six GO terms were significantly overrepresented (Table 3). These enriched annotations are consistent with known functions of Hoxa1, linking our set of interactors to developmental and transcription factor function. There were several additional enriched, though not statistically so, GO terms linked to development and transcription factors (Table 3).

**Table 3:** Gene Ontology (GO) enrichment analysis

**GO term**	Obs*	**Odds ratio**^**$**^	**P-value**^**£**^	**Corr P-value**^**μ**^
keratin filament	6	102,352	3,62292E-10	1,19194E-07
pattern specification process	6	12,3435	2,01548E-05	0,00331547
regionalization	5	14,3981	0,000048482	0,00531685
cranial nerve morphogenesis	2	116,248	0,000331888	0,0272978
kidney development	3	21,403	0,000559756	0,0306933
zinc ion binding	16	2,99882	0,000514454	0,0338511
embryonic development	6	5,58735	0,00120012	0,0564059
receptor signaling protein serine/threonine kinase activity	4	8,11769	0,00208634	0,0624006
developmental process	20	2,38882	0,00204634	0,0673246
negative regulation of MAP kinase activity	2	19,4953	0,00596027	0,0676182
regulation of transcription factor import into nucleus	2	31,8952	0,00251195	0,0688694
cation binding	19	2,14904	0,00649842	0,071266
anterior/posterior pattern formation	3	13,3725	0,00198155	0,0724365
cytoskeletal part	8	3,92527	0,00191605	0,0787977
inner ear morphogenesis	2	27,0115	0,00335617	0,0788701

* number of Hoxa1 interactors annotated with the corresponding GO term.

^$^ the odds ratio represents the enrichment of the corresponding GO term in the set of Hoxa1 interaction partners, an odds ratio of 10 meaning that the considered GO term is observed 10 times more than expected at random.

^£^ probability to see at least the number of proteins corresponding to the GO term at random.

^μ^P-value including a correction for multiple testing.

The immediate interactors of Hoxa1 were not enriched for annotated pathways, which could be due to incomplete coverage or relative sensitivity of the Y2H assay [35], or be intrinsic to the way Hoxa1 interacts with pathways, needing only one or few direct contacts. To account for the latter possibility, we also analyzed second-degree interactors, proteins that interact with Hoxa1 targets. Proteins associated with 21 pathways are overrepresented compared to random expectation (Table 4), showing that Hoxa1 could play a role in various processes other than gene regulation, such as focal adhesion, axon guidance or several signaling cascades.

**Table 4:** Pathways enriched in secondary Hoxa1 interactors

**Pathway name**	ID*	**Obs**^**$**^	**Odds ratio**^**£**^	**FDR**^**μ**^	**Corr FDR**^**§**^	**Source**	**Gene symbols**	**Entrez gene IDs**
RXR and RAR hetrodimerization with other nuclear receptor	pc926	2	24,83	2,00E-05	2,40E-03	NCI-Nature	FAM120B,NR1H2	84498,7376
Cell adhesion molecules (CAMs) - Homo sapiens (human)	hsa04514	2	19,70	9,00E-05	1,35E-02	KEGG	CLDN2,PVRL2	9075,5819
Gap junction - Homo sapiens (human)	hsa04540	2	16,59	8,00E-05	1,20E-02	KEGG	GNAI2,PDGFRB	2771,5159
Signaling events mediated by PTP1B	pc948	4	12,72	1,00E-05	1,20E-03	NCI-Nature	CRK,SPRY2,TRPV6,PDGFRB	1398,10253,55503,5159
Retinoic acid receptors-mediated signaling	pc960	3	10,19	2,10E-04	2,52E-02	NCI-Nature	NR1H2,NRIP1,FAM120B	7376,8204,84498
Integrins in angiogenesis	pc989	3	10,04	3,40E-04	4,08E-02	NCI-Nature	CDKN1B,SPP1,VCL	1027,6696,7414
Down-stream signal transduction	pc690	2	8,28	5,00E-05	3,14E-02	Reactome	PDGFRB,NCK2	5159,8440
Signaling by PDGF	pc876	2	8,28	5,00E-05	3,14E-02	Reactome	PDGFRB,NCK2	5159,8440
Toll-like receptor signaling pathway - Homo sapiens (human)	hsa04620	3	7,54	3,20E-04	4,80E-02	KEGG	TRAF6,TOLLIP,SPP1	7189,54472,6696
Homologous recombination - Homo sapiens (human)	hsa03440	2	7,18	1,40E-04	2,10E-02	KEGG	TOP3B,RAD54B	8940,25788
LPA receptor mediated events	pc1042	4	7,07	1,30E-04	1,56E-02	NCI-Nature	BIRC2,TRIP6,TRAF6,CRK	329,7205,7189,1398
Focal adhesion - Homo sapiens (human)	hsa04510	8	6,55	3,00E-05	4,50E-03	KEGG	SPP1,VCL,VASP,CRK,PDGFRB,BIRC2,CCND3,PAK7	6696,7414,7408,1398,5159,329,896,57144
TCR signaling in naive CD4+ T cells	pc1031	4	6,54	3,00E-04	3,60E-02	NCI-Nature	BIRC2,TRAF6,GRAP2,TRPV6	329,7189,9402,55503
Axon guidance - Homo sapiens (human)	hsa04360	5	6,50	1,40E-04	2,10E-02	KEGG	GNAI2,PAK7,NTN4,ABLIM1,NCK2	2771,57144,59277,3983,8440
TCR signaling in naive CD8+ T cells	pc997	4	6,34	3,80E-04	4,56E-02	NCI-Nature	TRAF6,GRAP2,TRPV6,BIRC2	7189,9402,55503,329
Lysine degradation - Homo sapiens (human)	hsa00310	3	6,28	1,00E-04	1,50E-02	KEGG	OGDH,AASDHPPT,EHMT2	4967,60496,10919
IGF1 pathway	pc1041	3	6,26	3,60E-04	4,32E-02	NCI-Nature	CRK,NCK2,YWHAE	1398,8440,7531
p75(NTR)-mediated signaling	pc978	5	6,02	2,60E-04	3,12E-02	NCI-Nature	YWHAE,TRAF6,BIRC2,RTN4,LINGO1	7531,7189,329,57142,84894
Proteogylcan syndecan-mediated signaling events	pc1045	5	5,53	2,20E-04	2,64E-02	NCI-Nature	BSG,HGS,CRK,SPRY2,SPP1	682,9146,1398,10253,6696
Syndecan-1-mediated signaling events	pc974	3	5,46	1,60E-04	1,92E-02	NCI-Nature	BSG,CRK,HGS	682,1398,9146
Plasma membrane estrogen receptor signaling	pc1048	6	4,07	3,60E-04	4,32E-02	NCI-Nature	CRK,SLC9A1,IRF4,NCK2,YWHAE,TRAF6	1398,6548,3662,8440,7531,7189

* pathway identifier.

$ number of Hoxa1 interactors belonging to the corresponding pathway.

^£^ the odds ratio represents the enrichment of the corresponding pathway, an odds ratio of 10 meaning that the considered pathway is observed 10 times more than expected at random.

^μ^false discovery rate as computed through the simulation.

^§^corrected false discovery rate accounting for multiple testing.

### Hoxa1-mediated interactions take place in distinct cell compartments

**Figure 2:**
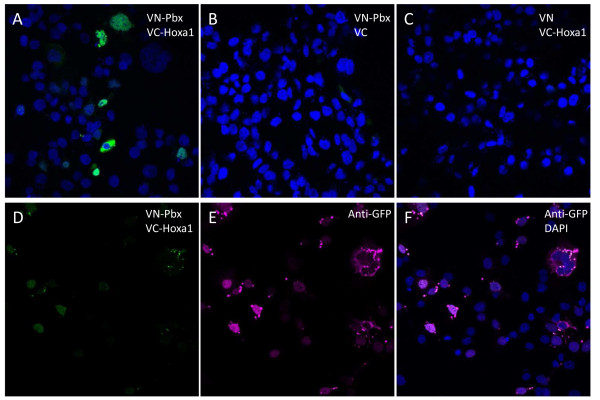
**Bimolecular Fluorescence Complementation assay reveals the Hoxa1-PBX1A interaction in culture cells.** COS7 cells were transfected with combinations of VN173, VN173-PBX1A, VC155 and VC155-Hoxa1 expression vectors. Upon interaction between PBX1A and Hoxa1, the VN173 and VC155 moieties of the Venus fluorescent protein brought together provide a fluorescent signal (**A**). Fluorescence complementation does not appear when the VN173 or VC155 fragments are expressed instead of the corresponding fusion proteins (**B**-**C**). As a control, expression of Venus fragments is detected by immunocytochemistry (anti-GFP). The BiFC signal shows colocalization with the anti-GFP immunofluorescence (**D**-**F**).

**Figure 3:**
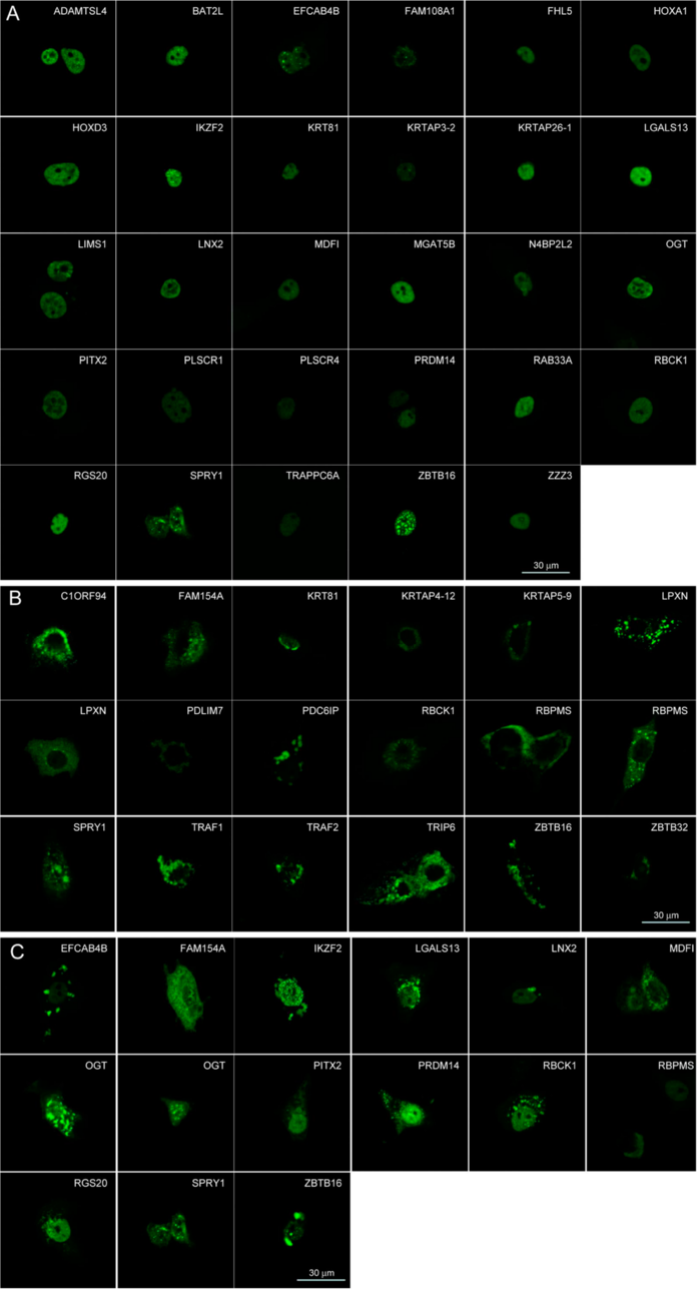
**Bimolecular Fluorescence Complementation assay reveals the Hoxa1-mediated interactions in culture cells.** MCF10A cells were transfected with VN173-hORF and VC155-Hoxa1 fusion proteins. Upon interaction between the partner proteins, the VN173 and VC155 moieties of the Venus fluorescent protein brought together provide a fluorescent signal. The interactions between Hoxa1 and its interactors can be classified according to their intracellular pattern: (**A**) nuclear, (**B**) cytoplasmic or associated to vesicles, (**C**) nuclear and cytoplasmic and/or vesicular.

**Figure 4:**
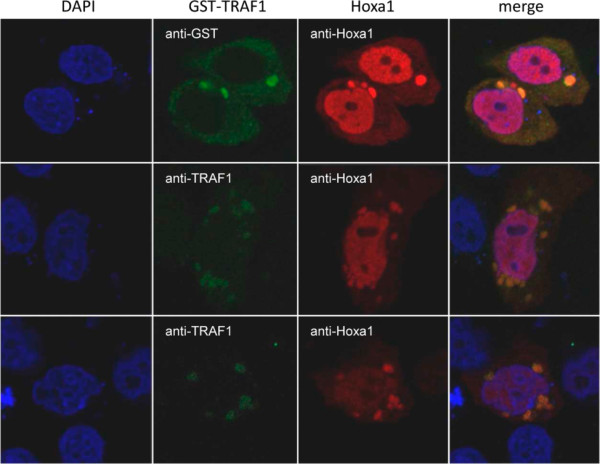
**Hoxa1 and TRAF1 intracellular distributions overlap.** MCF10A cells were transfected with GST-TRAF1 and Hoxa1 expression vectors. The immunolocalization of GST-TRAF1 and Hoxa1 (anti-GST, anti-TRAF1 and anti-Hoxa1 immunocytofluorescence) reveals that the partner proteins display partially overlapping intracellular distribution.

We tested the 45 validated Hoxa1 interacting proteins by Bimolecular Fluorescence Complementation (BiFC) assay, which not only tests for protein interactions but can also visualize where the distinct interactions occur in live cells. For BiFC, the ORF corresponding to each interactor was fused C-terminally to the N-terminal 173 amino acids of the Venus fluorescent protein (VN173), while the Hoxa1 ORF was fused downstream of the C-terminal moiety of Venus (amino acids 155 to 243; VC155). Detectable fluorescence in cells transfected for the complementary VN173 and VC155 fusion proteins means that a functional Venus has been reconstituted, indicating that the partner proteins interact. As a preliminary control, BiFC was assayed for the well-established Hoxa1-PBX1A interaction (Figure 2). The VN173-PBX1A and VC155-Hoxa1 fusion proteins provided fluorescence complementation (Figure 2A), whereas the VN173-PBX1A/VC155 and VN173/VC155-Hoxa1 combinations did not (Figure 2B, C). This therefore supported that the N- and C-terminal Venus fragments did not reassociate if not fused to interacting proteins. In addition, the immunocytolocalization of Venus consistently revealed that the VN173- and VC155-containing fusion proteins displayed a broad intracellular distribution that completely encompassed the narrower BiFC signal. In agreement with these controls, like the VN173-PBX1A fusion (Figure 2B), none of the VN173-interactor fusions provided fluorescence alone or in the presence of the VC155 Venus fragment alone (data not shown). For 41 out of the 45 interactors tested specific fluorescence was observed upon addition of the VC155-Hoxa1 fusion protein. Distinct patterns of intracellular interactions were observed (see Table 1 and 2, Figure 3). For 31 proteins, interactions took place in the nucleus (Figure 3A and C). Of these, 16 proteins appeared to contact Hoxa1 exclusively in the nucleus, while 15 also displayed other patterns of subcellular fluorescence complementation. Among the proteins found to bind Hoxa1 in the nucleus, some were known transcription factors (Table 5) or were known to have nuclear functions, but other were not (e.g. LGALS13, LIMS1, LNX2, MGAT5B, RBPMS, RAB33A, RGS20, TSCR1). A set of proteins shared a similar interaction pattern characterized by a diffuse, finely-punctuated cytoplasmic signal without nuclear staining (Figure 3B). This subcellular localization pattern was observed for different proteins reported to participate in a common signaling pathway. Examples are TRAF, TRIP or PDCD6IP (also known as Alix) which are found associated with the TNFR family of receptors [36-41], SPRY1 and PDCD6IP modulating RTK downstream signaling [42-46], PDLIM7 (alias LMP1) and RBPMS (also known as Hermes) which are involved in the BMP/TGFβ signaling regulation [47,48] and LPXN, PDCD6IP and TRIP6 known to associate with focal adhesion sites and related signal transduction [49-53]. As a control, in cells co-expressing GST-TRAF1 fusion and wildtype Hoxa1, proteins displayed an overlapping intracellular distribution consistent with the BiFC signal observed with VN173-TRAF1/VC155-Hoxa1 (Figure 4). Fourteen interactors tested displayed variable interaction patterns, showing mostly nuclear to nuclear and cytoplasmic or nuclear and vesicular BiFC signal (Figure 3A and C). This heterogeneous distribution suggests a coordinated shuttling between cell compartments for Hoxa1 and some partners (e.g. MDFI, OGT, PITX2, PRDM14, RBCK1, RBPMS, SPRY1, ZBTB16). The specific associations between Hoxa1 and 41 interactors detected by BiFC shows that Hoxa1 can associate dynamically with distinct categories of proteins in distinct intracellular domains.

**Table 5:** Functional classification of Hoxa1 interactors

**Function**	**Interactor**	**Function**	**Interactor**
**Cell shape and migration**		**Signal transduction** (continued)	
Focal adhesion associated	LIMS1 (PINCH1)	Other	MDFI
	LPXN		PDLMI7 (LMP-1)
	MGAT5B		PLZF
	PDCD6IP (Alix)		SMOC1
	TRIP6		
Cytoskeleton binding	FHL5 (ACT)	**Vesicular trafficking**	PDCD6IP
	LPXN		Rab33A
	PDCD6IP (Alix)		SPRY2
	TRIP6		TRAPPC6A
Cell junctions dynamics	LNX-2		TRIM23 (ARD1)
**Signal transduction**		**Transcription regulation**	FHL5 (ACT)
			GRN precursor
BMP/TGFb	PDLIM7 (LMP-1)		HOXA1
	RBPMS (Hermes)		HOXD3
Growth factors/RTK	LIMS1 (PINCH1)		HSFY1
	PDCD6IP (Alix)		IKZF2 (Helios)
	PLSCR1		LPXN
	SPRY2		MDFI (I-mfa)
	SPRY1		N4BP2L2
TNFR family	PDCD6IP (Alix)		OGT
	RBCK1		PITX2
	TRAF1		PLSCR
	TRAF2		PRDM14
	TRIM23 (ARD1)		RBCK1
	TRIP6		TRAPPC6A
Wnt/b-catenin	MDFI		TRIP6
Focal adhesion/integrin	LIMS1 (PINCH1)		ZBTB16 (PLZF)
	LPXN		ZBTB32 (FAZF)
	PDCD6IP (Alix)		ZZZ3
	TRIP6		
Notch	LNX-2	**Secreted protein/ECM**	EFEMP2
Protein kinases	LGALS13		GRN
	MDFI		PDCD6IP
	PLSCR1		SMOC1
	RBCK1		
	RGS20	**Miscellaneous**	KRT81
	SPRY2		KRTAP
	TRIP6		PLSCR
GPCR	RGS20		PLZF
	ZBTB16 (PLZF)		RAB33A
			RBPMS

## Discussion

By a high-throughput Y2H screen we identified 59 Hoxa1 interacting proteins among which 45 were confirmed by co-precipitation from animal cells. The intracellular localization of 41 interactions was further detected by a BiFC approach. This is the first exhaustive screen and analysis for interactors of a Hox protein. Our data support the conclusion that Hox proteins, and Hoxa1 in particular, known as crucial transcription factors controlling developmental processes can fulfill unexplored roles in cell signaling, cell adhesion, or vesicular trafficking.

Hoxa1 appears to interact with several proteins found to be part of molecular platforms associated with a few signaling pathways (TNFR superfamily, RTK, BMP/TGFβ, Focal adhesion,…), membrane dynamics and vesicular trafficking (Table 5). These platforms contact activated receptors at the plasma membrane and can positively or negatively modulate the downstream signaling or subsequent internalization in the endosomal compartment. By interacting with these proteins Hoxa1 could either act as a modulator or an effector of these signaling pathways. The BiFC assay revealed that most of the interactors involved in signaling pathways display a similar pattern of Hoxa1 interaction in culture cells. LPXN, PDLIM7, PDCD6IP, RBPMS, SPRY1, TRAF1, TRAF2 and TRIP6, for example, showed a BiFC signal in the cytoplasm, with fine punctuated staining probably related to vesicular compartments (Figure 2B). Although further experiments are required to identify these compartments, our data suggest that Hoxa1 interacts with distinct modulators of a given pathway at the level of shared molecular platforms. Finally, some interactors such as MDFI, OGT, RBCK1, RBPMS or SPRY1 display various patterns of Hoxa1 interaction from cell to cell, possibly indicating dynamic partnerships depending on cell physiological state (Figure 3A and C).

Some links might be drawn between the molecular, cellular and developmental processes involving Hoxa1 and its interactors. LIMS1 for example is expressed in neural crest cells and plays an important role in neural crest development through TGFβ signaling [54]; in mouse, a downregulation of SPRY1 inhibits the rhombomere4-derived neural crest cells to colonize the 2^nd^ branchial arch [55]; RBPMS is expressed in the outflow tract of the developing heart [56], a territory colonized by Hoxa1 positive cells [57]. An important group of interactors consists in transcription factors. Some of them are known to be involved in embryonic patterning or cell fate decision (HOXD3, MDFI, PITX2 for example). In that regard, ZBTB16 (better known as PLZF) is a particularly relevant Hoxa1 interactor. It is expressed during hindbrain development at rhombomere boundaries and, like Hoxa1, has been proposed to control hindbrain segmentation [58]. Transcriptional coregulators, like the SET-domain histone methyl-transferase PRDM14 or the O-linked-N-acetyl-glucosamine (GlcNac) transferase OGT, have also been identified as Hoxa1 interactors which may contribute to Hoxa1-mediated gene regulation. Most significantly, OGT has recently been shown to be the homologue of the Drosophila Super sex combs (Sxc) protein. Sxc is associated to Polycomb complexes and is required for their ability to repress gene expression, including *Hox* genes [59].

## Conclusions

We presented here the first large-scale Hox interactome characterized so far. Although only a handful of interactors are known for other Hox proteins, some interactors identified here for Hoxa1 are shared with other Hox proteins [28]. PLSCR1 has been shown to contact HOXA9 and HOXB6, and HOXA9 is also contacted by TRIP6. RBPMS is able to interact with HOXA9 and HOXB9. These interactions, as well as other described here, underline that Hox proteins should be viewed not only as gene regulators, but also as components of signal transduction and modulation of cell-to-cell communication, cell adhesion and vesicular trafficking.

## Methods

### Yeast two-hybrid screening

The mouse *Hoxa1* coding sequence was amplified from the pGIH327 expression plasmid[60] and cloned into pDONR-223 by Gateway BP recombinational reaction (*att*B1.1 primer: *GGGGACAACTTTGTACAAAAAAGTTGGCATG*AACTCCTTTCTGG; *att*B2.1 primer: *GGGGACAACTTTGTACAAGAAAGTTGGGTA*GTGGGAGGTAGTCAGAGTGTC; Invitrogen). By Gateway LR recombinational cloning, *Hoxa1* was then transferred into pDEST-DB and pDEST-AD-CYH2 centromeric destination vectors [29] to code for Gal4 DNA binding domain (DB)-Hoxa1 and Gal4 activation domain (AD)-Hoxa1 fusion proteins, respectively.

*MAT***α** Y8930 and *MAT***a** Y8800 yeast strains (genotype: *trp1-901; leu2-3, 112; ura3-52; his3-200; gal4Δ; gal80Δ; GAL2-ADE2; LYS2::GAL1-HIS3; met2::GAL7-lacZ; cyh2*^*R*^) were used for yeast two-hybrid (Y2H) screens. The DB-Hoxa1 coding construct was first tested for auto-activation by transforming it into the *MAT***α** Y8930 yeast strain and testing for expression of the *HIS3* reporter gene in the absence of any AD-hORF fusion protein, on a solid synthetic complete medium lacking leucine and histidine (Sc-L-H) and supplemented with 1mM 3-amino-triazol (3AT) [29]. The DB-Hoxa1 construct did not auto-activate.

High-throughput Y2H screens were essentially performed as described [29]. Briefly, DB-Hoxa1 and AD-Hoxa1 vectors were transformed into *MAT***α** Y8930 or *MAT***a** Y8800 yeast strains, respectively. The DB-Hoxa1 construct in *MAT***α** Y8930 was mated with *MAT***a** Y8800 containing the AD-hORF library [27], and for the other configuration DB-hORFs library in *MAT***α** Y8930 were mated with AD-Hoxa1 in *MAT***a** Y8800. After overnight growth at 30°C, diploid yeast cells were transferred to plates lacking histidine, leucine and tryptophan, supplemented with 1mM 3AT (Sc-L-T-H+3AT), to select for those with elevated expression of the *GAL1-HIS3* reporter gene.

Positive colonies were picked, grown on Sc-L-T plates, and retested on Sc-L-T-H, as well as on medium lacking Adenine (Sc-L-T-A) and Sc-L-T-H-A+3AT, to select for colonies with high *GAL1-HIS3* and *GAL2-ADE2* reporter gene activity. To detect any spontaneous auto-activators arising in the course of the screen, positive colonies were transferred in parallel onto cycloheximide containing media (Sc-H+CHX). Candidate colonies that grew on Sc-H+CHX were discarded.

The identities of candidate interacting pairs was determined by sequencing PCR products amplified directly from yeast cells using primers specific to Gal4DB and Gal4AD (DB primers: *GGCTTCAGTGGAGACTGATATGCCTC*, *GGAGACTTGACCAAACCTC TGGCG*; AD primers: *CGCGTTTGGAATCACTACAGGG*, *GGAGACTTGACCAAACC TCTGGCG*). PCR products were purified (Qiagen kit # 28104) and sequenced.

The protein interactions from this publication have been submitted to the IMEx (http://www.imexconsortion.org) consortium through IntAct [pmid: 19850723] and assigned the identifier IM-15418.

### Co-precipitation assays

The *Hoxa1* coding sequence was transferred from the pDONR-223 Gateway® vector to pDEST-FLAG mammalian expression vector by Gateway® LR recombination reaction. Open reading frames coding for interactors from the hORFeome were cloned into a pDEST-GST mammalian expression vector by the same procedure.

COS7 and HEK293T cells were maintained in Dulbecco’s modified Eagle’s medium (DMEM) low glucose or high glucose respectively (Gibco/Invitrogen) supplemented with Glutamine, 10% fetal bovine serum (Gibco/Invitrogen), 100 IU/ml penicillin, and 100 μg/ml streptomycin (Gibco/Invitrogen). Cell lines were maintained at 37°C in a humidified, 5% CO_2_ atmosphere. For transient transfection, 1.4 × 10^5^ (COS7) or 4 × 10^5^ (HEK293T) cells were plated into six-well plates. Twenty-four hours after plating, cells were transfected with TransFectin™ reagent (BioRad). One and a half μg of pDEST-FLAG-Hoxa1 expression vector and 3μg of pDEST-GST-hORF were mixed with 250μl of serum-free medium and added to a mix of 1 μl of TransFectin™ and 250μl of serum-free medium. Forty-eight hours after transfection, cells were lysed with Tris–HCl pH7.5 20mM, NaCl 120mM, EDTA 0.5mM, NP40 0.5%, glycerol 10% and Complete™ protease inhibitor (Roche).

Cell lysates were cleared by centrifugation for 5 minutes at 13,000 g. Cleared lysates were incubated overnight on gluthatione-agarose beads (Sigma # G4510). Beads were cleared 3 times with the lysis buffer. Beads and third wash samples were then loaded on SDS-PAGE, transferred on nitrocellulose membrane and processed for detection of FLAG tagged proteins with an anti-FLAG M2 antibody (Sigma # F1804).

### Bimolecular Fluorescence Complementation assay (BiFC)

pDEST-VN173 and pDEST-VC155 plasmids were obtained by cloning sequences encoding N-terminal residues 1–173 and C-terminal residues 155–243 of the yellow fluorescent protein VENUS, respectively, within the pDEST-v1899-FLAG vector instead of the 5’ [K*pn*I|H*ind*III] 3xFLAG-fragment (VN173F primers : *GAGGTACCATGGTGAGCAAGGGCGAGGAGC*, *GGAGAAGCTTCTCGATGTTGTGGCGGATC*; VC155 primers: *AAGGTACCATGGCCGACAAGCAGAAGAACGGC*, *GGAAAAGCTTCGTGGACCGGTGCTTGTACAGC*).

The *Hoxa1* coding sequence was transferred from the pDONR-223 Gateway® vector to pDEST-VC155 mammalian expression vector by Gateway® LR recombination reaction. Open reading frames coding for interactors from the hORFeome were cloned into the pDEST-VN173 mammalian expression vector by the same procedure.

MCF10A cells were maintained at 37°C in a humidified 5% CO_2_ atmosphere, in DMEM-F12+L-glutamine medium (Gibco/Invitrogen) supplemented with 5% horse serum (Gibco/Invitrogen), 100 IU/ml penicillin (Gibco/Invitrogen), 100 μg/ml streptomycin (Gibco/Invitrogen), 100 ng/ml of cholera toxin (Gentaur), 20 ng/ml of human Epidermal Growth Factor (hEGF; Sigma), 500 ng/ml hydroxycortisone (Sigma) and 10 μg/ml insulin (Sigma). For transfection, 3 × 10^5^ cells were seeded on glass cover slips in 24-well plates. Twenty-four hours after plating, cells were transfected with TransFectin™ reagent (BioRad) or JetPRIME (Polyplus). For JetPRIME transfection, a total of 500 ng of plasmid DNA were transfected per well: 100 ng of pDEST-VN173-hORF, 20 ng of pDEST-VC155-Hoxa1 and 380 ng carrier DNA. DNA was mixed with 50 μl JetPRIME buffer and 1 μl of JetPRIME was added further. For TransFectin™-mediated transfection, 500 ng of pDEST-VN173-hORF and 500 ng of pDEST-VC155-Hoxa1 were mixed with 50 μl of serum-free medium and added to a mix of 1 μl of TransFectin™ and 50 μl of serum-free medium. Twenty-four hours after transfection, cells were fixed with 4% formaldehyde for 30 minutes, rinsed three times in PBS and once in TBS-0,1% Triton X100. Glass cover slips were mounted in Vectashield®-DAPI medium (Vector laboratories). BiFC were then analysed by confocal microscopy (LSM710, Zeiss, Jena, Germany; Plan-Apochromat 63x/1.40 Oil DIC M27 objective; Oil refraction index 1.5 imaging medium; PMT camera). Images were acquired by using the ZEN 2010 software, and subsequently processed with ZEN 2008 Light Edition.

### Immunocytolocalization

COS7 and MCF10A cells were maintained, seeded on coverslips and transfected as described here above. Twenty four hours after transfection, cells were fixed with 4% formaldehyde for 30 minutes. Cells were further blocked with 10% low-fat milk in TBS-0.1% Triton X100 solution for 45 min at room temperature, followed by overnight incubation in TBS-0.1% Triton X100 solution at 4°C, with a rabbit polyclonal anti-GFP (Invitrogen A11122, diluted 1/200), a mouse anti-GST (Sigma G1160, diluted 1/50), a mouse monoclonal anti-TRAF1 (Santa Cruz, sc-6253, diluted 1/50), or a rabbit polyclonal anti-Hoxa1 (Abcam ab64941, diluted 1/50), as primary antibodies. Cells were rinsed three times for 30 min in TBS-0.1% Triton X100 solution and incubated for 45 min at room temperature with a goat anti-rabbit IgG-AF555 (Molecular Probes 4413, diluted 1/750), a goat anti-mouse IgG-FITC (SantaCruz sc-3699, diluted 1/100), or a bovine anti-rabbit IgG-TRITC (SantaCruz sc-2367, diluted 1/100), as secondary antibodies. Cells were rinsed three times and glass cover slips were mounted in Vectashield®-DAPI medium (Vector laboratories). Slides were then analysed by confocal microscopy (LSM710, Zeiss, Jena, Germany; Plan-Apochromat 63x/1.40 Oil DIC M27 objective; Oil refraction index 1.5 imaging medium; PMT camera). Images were acquired by using the ZEN 2010 software, and subsequently processed with ZEN 2008 Light Edition.

### Gene Ontology annotation and pathway analysis

Gene Ontology (GO) annotations were downloaded from Entrez Gene (September 2009), pathway data from KEGG (September 2008) and Pathway Commons (September 2008) databases. From Pathway Commons, we analyzed the pathways originally annotated in NCI-Nature[pid.nci.nih.gov] and Reactome [61].

Fisher’s Exact Test was used to determine GO annotation and pathway enrichment of Hoxa1 direct targets, using the space of human proteins that have been tested in our Y2H experiment, the human ORFeome v3.1 [27]. The corrected *p*-value was computed using the Benjamini-Hochberg multiple testing correction. We limited our results to GO annotations and pathways for which at least two Hoxa1 targets were annotated for.

To estimate the significance of indirect targets enrichment we ran 100,000 simulations for which the identity of the direct targets was randomized. The interactors of these targets were identified in an unbiased protein-protein interaction network [28], to avoid study bias inherent to literature curation. Interactors belonging to each pathway were counted, and the resulting distribution compared to the observed counts. An empirical False Discovery Rate (FDR) determined the significance of the enrichment, with the FDR computed as the proportion of random trials giving at least the observed number of indirect targets in the analyzed pathway. The FDR was corrected for multiple testing using the Bonferroni correction. Pathways with a corrected FDR < 0.05 and at least two observed proteins were considered significant.

## Abbreviations

TALE: Three Amino acid Loop Extension; TBP: TATA Binding Protein; HMG: High Mobility Group; CBP: CREB Binding Protein; Y2H: Yeast two-hybrid; BMP: Bone Morphogenetic Protein; TGF: Tumor Growth Factor; TNF: Tumor Necrosis Factor; RTK: Receptor Tyrosine Kinase; BiFC: Bimolecular Fluorescence Complementation; DB DNA: Binding domain; AD: Activation Domain; 3AT: 3-Amino-Triazol; GO: Gene Ontology; FDR: False Discovery Rate; ORF: Open Reading Frame; GST: Glutathione S-Transferase; NCoR: Nuclear receptor Co-Repressor; SMRT: Silencing Mediator of Retinoic acid and Thyroid hormone receptor; HDAC: Histone Deacetylase; KAP: Keratin Associated Protein.

## Competing interests

The authors declare that they have no competing interests.

## Authors' contributions

BL carried out most of the molecular biology, yeast two-hybrid and cell biology experiments, made a substantial contribution to data analysis and drafted the manuscript. JV contributed to the co-precipitation experiments and substantially contributed to the BiFC assay and immunocytofluorescent detection of proteins. SR and IB set up the BiFC assay and the BiFC controls. NS carried out the bioinformatics analyses. JCT helped in the yeast two-hybrid screening and data interpretation. MV conceived and provided the materials required for the high-throughput yeast two-hybrid assay. RR conceived the study, significantly contributed to data interpretation and helped in drafting and revising the manuscript. All authors read and approved the final manuscript.
